# Therapeutic potential of pomegranate juice-derived nanovesicles in nude mouse benign prostatic hyperplasia (BPH) xenograft model

**DOI:** 10.1038/s41598-023-39511-w

**Published:** 2023-08-01

**Authors:** Amritha Sreekumar, Matthew N. Simmons, Tae Jin Lee, Ashok Sharma, Sharanjot Saini

**Affiliations:** 1 Department of Biochemistry and Molecular Biology, Augusta University, 1410 Laney Walker Boulevard, Augusta, GA 30912 USA; 2 Department of Urology, Augusta University, Augusta, USA; 3 Department of Center for Biotechnology and Genomic Medicine, Augusta University, Augusta, USA

**Keywords:** Drug discovery, Diseases, Urology

## Abstract

Benign prostatic hyperplasia (BPH) and associated lower urinary tract symptoms affect a large percentage of the male population and places a substantial burden on the world health system. Current therapies include 5-alpha reductase inhibitors and alpha-blockers that are only partially effective and pose a huge economic burden, emphasizing the urgent need for effective, economical therapies. We isolated nanovesicles from pomegranate juice (*Punica Granatum*) (referred to as ‘POM-NVs’) and report to our knowledge for the first time, that these vesicles possess therapeutic potential against BPH. Following extensive characterization of POM-NVs, we tested their therapeutic potential in vitro using BPH1 cell line and identified a potential anti-proliferative and pro-apoptotic effect. We further tested these vesicles using a clinically relevant xenograft mouse BPH model derived from human BPH tissues. Remarkably, POM-NVs could reverse the BPH phenotype conferred by TGF-β mediated signaling and induced epithelial-to-mesenchymal (EMT) reversal, leading to the restoration of prostate epithelial states in vivo and in vitro. Furthermore, these vesicles attenuated bone morphogenic protein 5 (BMP5) signaling, a cardinal alteration that is instrumental in driving BPH. Considering the large incidences of BPH and its associated economic burdens, our study has important implications and can potentially improve the clinical management of BPH.

## Introduction

Benign prostatic hyperplasia (BPH) is a condition characterized by the gradual enlargement of the prostate gland's transition zone. This enlargement occurs due to excessive growth of the epithelial and stromal cells within the prostate. As a result, men experience lower urinary tract symptoms (LUTS) caused by obstruction of the bladder outlet^1–3^. The prevalence of BPH is widespread, with the disease incidence increasing by 10% every decade and approximately 50% of men aged 50 years are diagnosed with histologic BPH^4^. Symptoms include increased urinary frequency and urgency to acute urinary retention. In addition to these symptoms, serious complications frequently occur such as renal failure, bladder stones, urinary bleeding and infections.

The pathophysiology of BPH has been associated with androgen signaling, reactive stroma and inflammation^5,6^. In the normal development of the prostate gland, androgens primarily influence the stroma, which then regulates the proliferation and differentiation of prostatic epithelial cells^6,7^. As individuals age, these stromal signals can be reactivated, contributing to the pathogenesis of BPH^6,8^. Additionally, heightened inflammation associated with metabolic syndrome has been identified as a factor that can impact the prostatic stroma and is considered a determining factor in prostate enlargement^9^. According to recent genomic analyses of BPH, it has been discovered that this condition is not simply characterized by prostatic hyperplasia. Instead, there is a distinct depletion and enrichment of specific prostate cell types found in both the BPH epithelium and stroma. Notably, there is a reduction in neuroendocrine cells and a particular fibroblast cell type that expresses estrogen receptors near the epithelium, while there is an increase in a subset of myofibroblasts^6^. BPH has been associated with decreased E-cadherin expression and accumulation of mesenchymal-like cells derived from the prostatic epithelium and the endothelium, changes consistent with epithelial-to-mesenchymal transition (EMT)^10–12^. It has been shown that TGF-β mediates EMT in BPH via activation of Smad signaling^12^. An elevation in bone morphogenic protein 5 (BMP5) signaling has been reported to be a cardinal alteration driving BPH^6^. A member of the TGF-β family, BMP5 promotes cell proliferation and EMT via phosphorylation of Smad1/5/8 in BPH^13^. While substantial advancements have been achieved in comprehending the pathobiology of BPH, we still have a long way to go in attaining a comprehensive understanding of the signaling pathways that underlie this condition. The available treatment options for BPH primarily focus on addressing the physiological aspects of the entire prostate rather than directly targeting the underlying mechanisms of the disease^6^. Currently, medical agents such as 5-alpha reductase inhibitors, which inhibit androgen-mediated growth, and alpha blockers, which relax the smooth muscle tone of the prostate and bladder neck, are utilized. While these therapies reduce the risk of symptomatic progression by 30–40%^9^, there is a pressing need for more effective treatments due to their limited efficacy. Moreover, the current therapies impose a significant economic burden.

Nanovesicles, measuring approximately 30–150 nm in diameter, consist of a lipid bilayer and contain a diverse assortment of bioactive molecules such as proteins, lipids, nucleic acids (RNA and DNA), and metabolites^14^. These small vesicles are secreted by most cell types and play a crucial role in intercellular communication. These vesicles encompass small vesicles of various origins and biogenesis pathways, with exosomes being one of them. Exosomes are a type of membranous extracellular vesicles (EVs), which are nano-sized structures (30–150 nm) released by all living cells, including bacteria, plants, and eukaryotes^15^. They are formed within the endosomal compartment of cells through the inward budding of the limiting membrane of multivesicular bodies (MVBs). Following fusion of these MVBs with the cell's plasma membrane, the internal vesicles are released and referred to as "exosomes". The primary function of exosomes range from disposing of toxic materials to facilitating intercellular communication by delivering their cargo, including mRNA and proteins, to recipient cells to influence their functions^16,17^. Extensive research has been conducted on mammalian nanovesicles, revealing their involvement in various physiological and disease processes^18^. Due to their capacity to transport a diverse range of molecules, nanovesicles have been effectively utilized in the treatment of different diseases. Plant-derived nanovesicles, on the other hand, have not received much attention. In 2009, plant EVs were first isolated from plant apoplastic fluid and observed using Transmission Electron Microscopy (TEM)^19,20^. In this study, we successfully isolated nanovesicles from pomegranate juice (*Punica Granatum*) and, to the best of our knowledge, provide the first evidence of their therapeutic potential against BPH. Pomegranate was chosen as the source of these vesicles due to its recognized beneficial effects on prostatic health. Extensive research has demonstrated the chemopreventive and chemotherapeutic properties of pomegranate juice in prostate cancer^21^. Pomegranate is rich in polyphenols called anthocyanidins, which have been found to inhibit cell proliferation and induce apoptosis in prostate, breast, and pancreatic cancer cell lines by mobilizing intracellular copper ions and generating reactive oxygen species^22,23^. Additionally, a higher consumption of dietary anthocyanidins has been associated with a lower risk of renal cancer^24^. These studies emphasize the potential of pomegranate in cancer prevention and treatment.

However, the effects of pomegranate on BPH are not well understood. One study examined the effects of orally administered pomegranate fruit extract (PFE) on testosterone-induced BPH in rats. They reported that PFE reduced COX-II expression, decreased Ki67 staining, and induced apoptosis^25^. However, all previous mechanistic investigations have utilized pomegranate "extract" preparations or purified polyphenols, which have limitations in terms of clinical applicability due to their inability to exert direct effects when administered systemically. Concerns include potential toxicity and non-specific activity. To address these limitations, we have developed a robust protocol to isolate nanovesicles from pomegranate juice, referred to as POM-NVs, and have evaluated their effectiveness as therapeutic agents for BPH. Significantly, these vesicles demonstrated the ability to diminish BMP5 signaling and counteract the effects of TGF-β on epithelial-to-mesenchymal transition (EMT), thereby restoring the prostate epithelial states in a clinically relevant BPH model derived from human BPH tissues. Our findings strongly support the promising potential of these novel nanovesicles derived from pomegranate (POM-NVs) as an effective treatment option for BPH. Notably, since these nanovesicles originate from a dietary source, they offer an economical, non-toxic, and scalable solution. Our study has important translational implications and can lead to better clinical management of BPH.

## Results

### Isolation and characterization of pomegranate juice-derived nanovesicles

**Figure 1:**
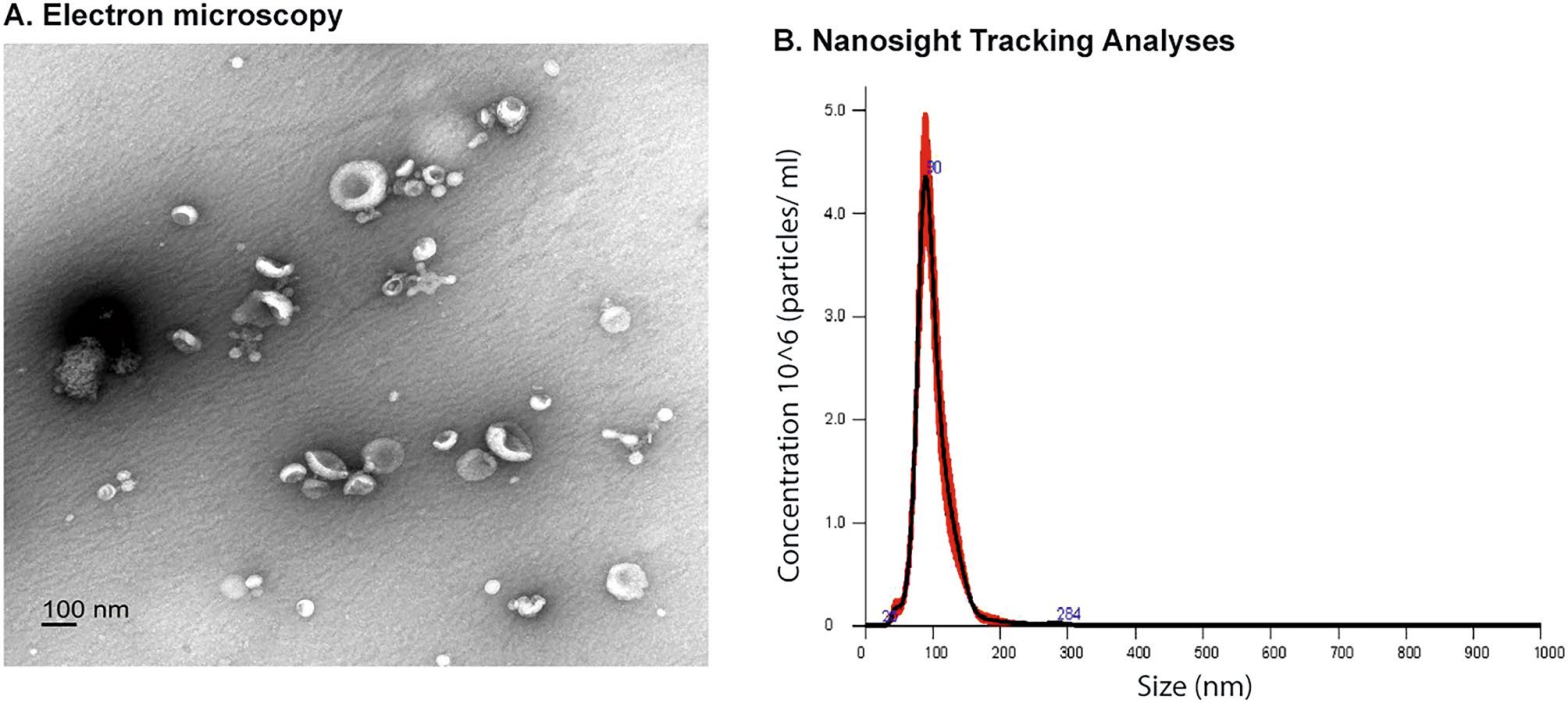
Characterization of pomegranate juice-derived nanovesicles. (**A**) Transmission electron microscopy of POM-NVs. Scale bar 100 nm. (**B**) Nanoparticle Tracking Analyses (NTA) of POM-NVs showing size and concentration of isolated particles.

With our goal of identifying economical, effective and safe therapies for BPH, we examined the potential of dietary nanovesicles. Given the well-documented positive effects of pomegranate on prostatic health, we successfully isolated nanovesicles from pomegranate juice, which we referred to as POM-NVs, utilizing a combination of ultracentrifugation and filtration techniques. To ensure their characterization, we extensively examined these vesicles using transmission electron microscopy (TEM) (Fig. 1A) and Nanosight Tracking Analysis (NTA) (Fig. 1B). The TEM analysis revealed cup-shaped vesicles within the size range of 100–200 nm. Furthermore, NTA confirmed the isolation of vesicles of approximately 100–200 nm in size, with a concentration of approximately 1.6 × 10^9^ particles/ml of pomegranate juice.

### Nanovesicles derived from pomegranate juice reduce viability and induce apoptosis in BPH1 cells

**Figure 2:**
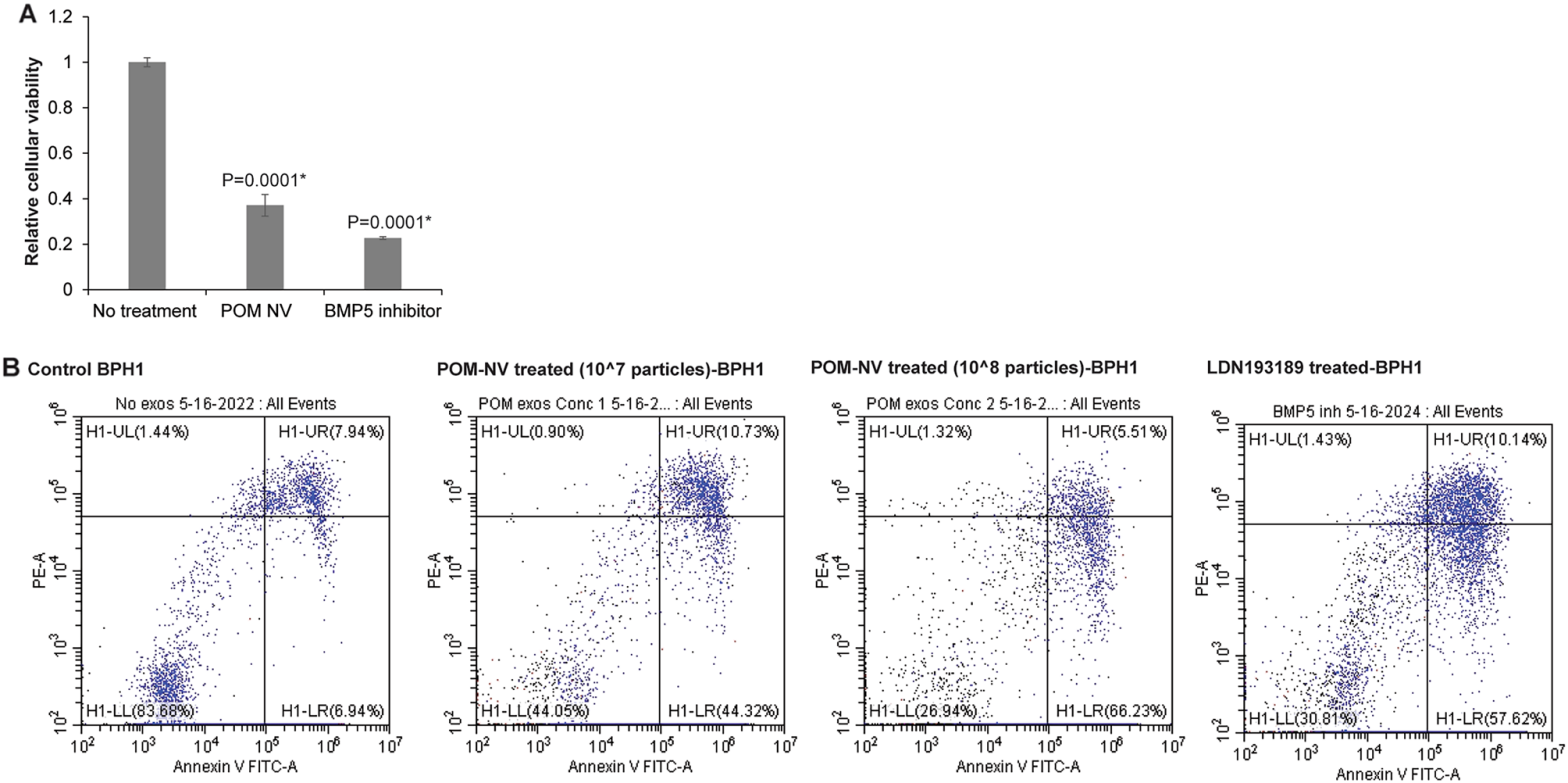
Nanovesicles derived from pomegranate juice reduce viability and induce apoptosis in BPH1 cells. (**A**) Relative cellular viabilities in control, POM-NVs treated (10^7^ particles) and BMP5 inhibitor (LDN 193189) treated BPH1 cells as assessed by MTS assay (post 48 h of treatments). (**B**) Annexin-V-FITC/7-AAD staining of BPH1 cells with no treatment control (left panel), BPH1 cells treated with 10^7^ POM-NVs or 10^8^ POM-NVs (middle panels) or LDN193189 (right panel).

To assess the therapeutic potential of POM-NVs, we conducted in vitro experiments using the immortalized BPH1 cell line^26^. The BPH1 cells were cultured in media depleted of exosomes and treated with POM-NVs, followed by functional assays (Fig. 2). As a positive control, we also treated BPH1 cells with LDN 193189, a promising BMP-5 inhibitor in BPH^13^. The MTS assay (Fig. 2A) revealed that POM-NVs significantly reduced the cellular viability of BPH1 cells compared to the control cells, similar to the effect observed with the BMP5 inhibitor treatment.

Furthermore, Annexin-V-FITC/7-AAD staining was performed on control BPH1 cells and BPH1 cells treated with increasing concentrations of POM-NVs (10^7^ or 10^8^ particles). The results showed that POM-NVs induced significant apoptosis, with the populations of early apoptotic and late apoptotic cells increasing from 6.94% + 7.94% in the control group (Fig. 2B, left panel) to 44.32% + 10.73% and 66.23% + 5.51% in BPH1 cells treated with 10^7^ particles and 10^8^ particles, respectively (Fig. 2B, middle panels), accompanied by a reduction in viable cells. This level of apoptosis induction was comparable to that achieved with the BMP5 inhibitor treatment (Fig. 2B, right panel). These findings suggest a potential anti-proliferative and pro-apoptotic effect of POM-NVs in BPH.

### Therapeutic effects of pomegranate juice-derived nanovesicles in BPH xenograft model

Considering the observed anti-proliferative and pro-apoptotic effects of POM-NVs on BPH1 cells in vitro, we proceeded to investigate their potential effects in vivo. To assess the potential of POM-derived nanovesicles in treating BPH in an in vivo setting, we established a xenograft mouse BPH model. With informed consent, human BPH tissues (transition zone) were obtained from patients through transurethral resection of the prostate (TURP). Subsequently, nu/nu mice underwent bilateral orchiectomy and received testosterone pellets ventrally. The collected BPH tissues were sliced and subcutaneously implanted under the dorsal skin. After allowing the xenografts to establish for 4 weeks, the mice were categorized into control and test groups (n = 4/group). The test group received 10^9^ POM-NVs intravenously via the tail vein twice a week for 3 weeks, while the control group received PBS treatment. After 3 weeks, the mice were sacrificed, and the xenografts were harvested.

**Figure 3:**
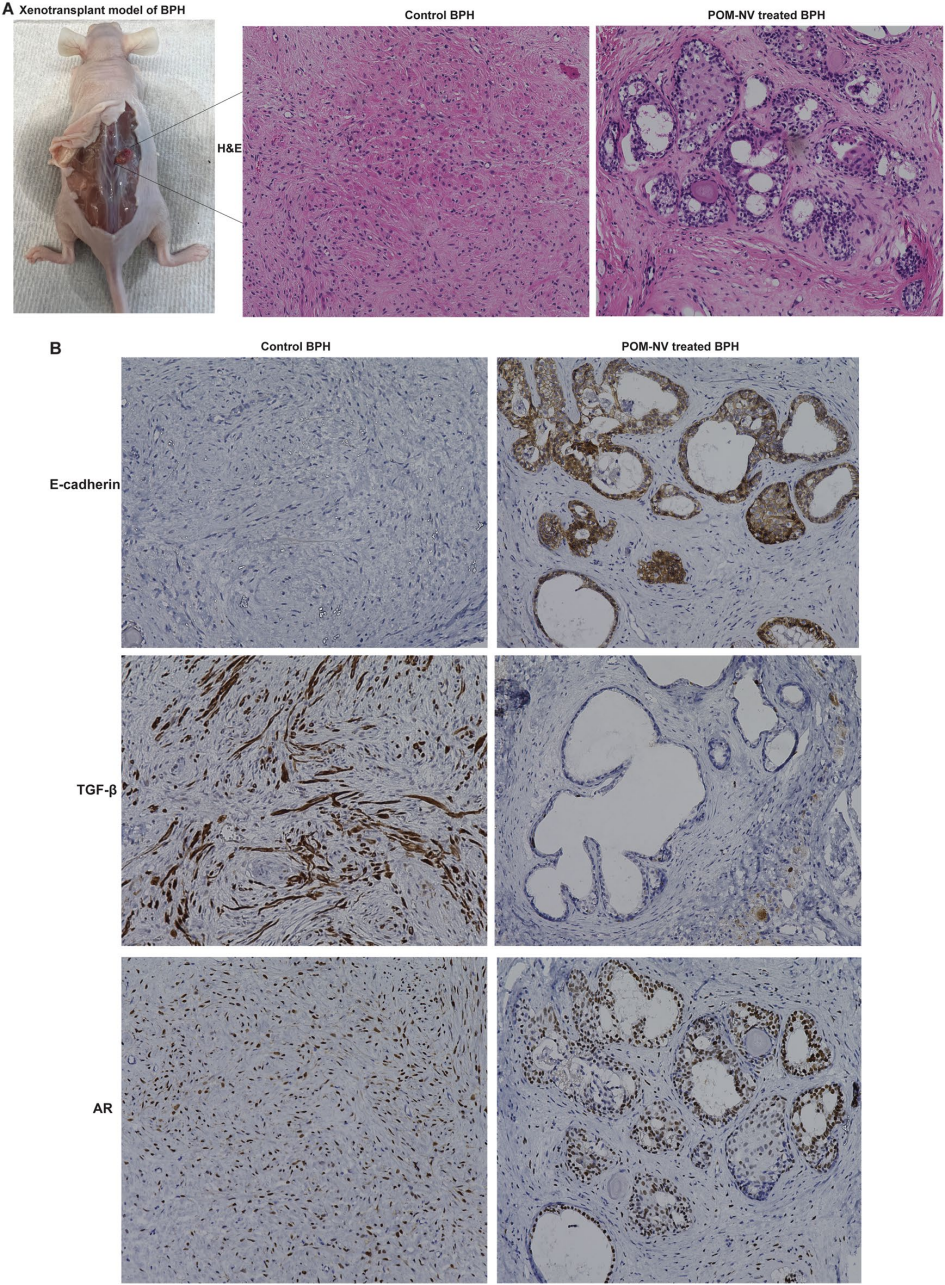
Therapeutic effects of pomegranate juice-derived nanovesicles in BPH xenotransplant model. (**A**) Xenograft model of BPH: Human BPH tissues (transition zone) were collected from patients by transurethral resection of the prostate (TURP). *nu/nu* mice underwent bilateral orchiectomy and placement of a testosterone pellet ventrally. BPH tissues were sliced into 1 mm sections (4 per mouse), covered in matrigel and implanted subcutaneously under the dorsal skin. Xenografts were allowed to establish for 4 weeks. After 4 weeks, xenografts were harvested. Right panels show H&E staining of control/POM-EV treated xenografts. (**B**) Xenografts from human clinical BPH tissues were established followed by control/POM-NVs treatment. Test mice were administered 10^9^ POM-derived nanovesicles via tail vein (in PBS) twice a week for 3 weeks. Controls included xenografts treated with PBS for the same time period at the same frequency. After 3 weeks, mice were sacrificed and xenografts were harvested. Top panels show E-cadherin staining of the xenografts, middle panels show IHC for TGF-β and lower panels show IHC for AR in control/POM-NVs treated groups.

Histological examination through hematoxylin and eosin (H&E) staining in the control group confirmed the viability of the xenografts and exhibited preserved prostate tissue architecture (Fig. 3A, left panel). In contrast, the POM-NVs-treated group displayed altered morphology with an increased presence of epithelial and glandular cells compared to the control group (Fig. 3A, right panel). Other histological changes included reduced stromal component, overall decreased cellularity, and areas of vacuolization following POM-NVs treatment. As BPH has been associated with TGF-β-mediated epithelial-to-mesenchymal transition (EMT)^12^, we performed immunohistochemical (IHC) staining for E-cadherin and TGF-β (Fig. 3B). Interestingly, E-cadherin staining demonstrated significantly increased expression upon POM-NVs treatment, showing persistent glandular staining and increased stromal staining compared to the control group (Fig. 3B, upper panels). TGF-β staining in the control group displayed significant stromal positivity in cells with or without a mesenchymal phenotype. However, in the POM-NVs-treated group, there was a consistent decrease in TGF-β expression, with significant reductions observed in mesenchymal cells and increased leukocytic TGF-β staining compared to controls (Fig. 3B, middle panels). IHC staining with a human androgen receptor (AR) antibody confirmed the preservation of AR staining in the xenografts, confirming their prostatic origin. Upon POM-NVs treatment, AR staining localized to a greater extent in epithelial and glandular cells (Fig. 3B, lower panels). These findings suggest that POM-NVs have the ability to reverse the BPH phenotype mediated by TGF-β signaling and induce EMT reversal.

### Pomegranate derived nanovesicles inhibit BMP5 signaling in BPH1 in vitro and in vivo

**Figure 4:**
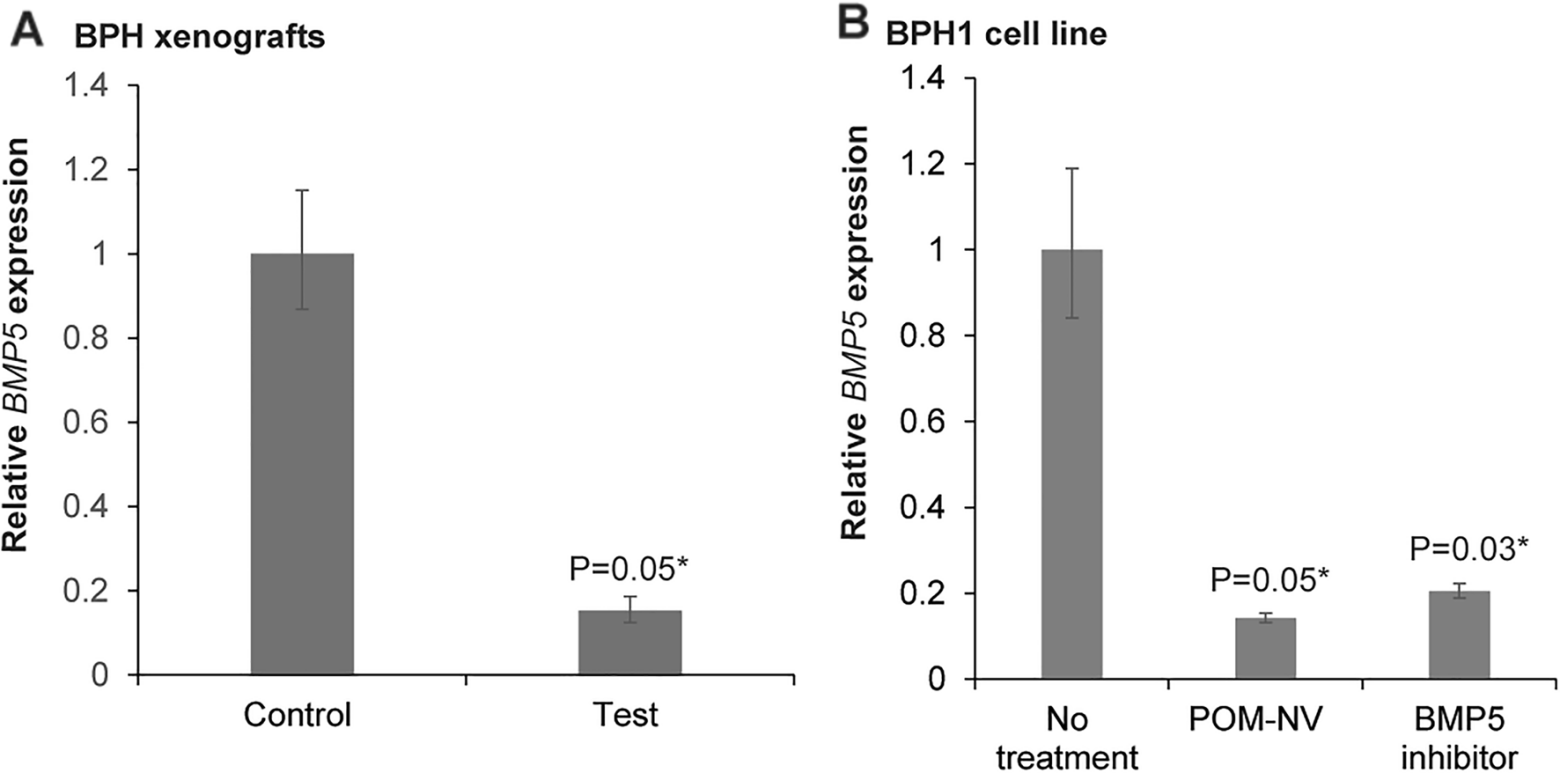
Pomegranate juice derived nanovesicles inhibit BMP5 signaling in BPH1 in vitro and in vivo. (**A**) Real time PCR based analyses of relative *BMP5* expression in control/POM-NVs treated BPH xenografts. Test mice were administered 10^9^ POM-derived nanovesicles via tail vein (in PBS) twice a week for 3 weeks while control mice received PBS for same duration. (**B**) Real-time PCR based analyses of relative *BMP5* expression in BPH1 cells treated with POM-NVs (10^8^ particles) for 2 days. As a positive control, BPH1 cells were treated with BMP-5 inhibitor LDN 193189 for 2 days. GAPDH was used as an endogenous control.

Given the observed therapeutic effects of POM-NVs in both in vitro and in vivo experiments, we investigated their impact on BMP5 signaling, as upregulated BMP5 signaling has been implicated as a key factor driving BPH^6^. Tissues from the in vivo BPH xenograft model (Fig. 3) were harvested, and real-time PCR was performed to analyze relative BMP5 expression (Fig. 4A). Interestingly, we observed a remarkable decrease in BMP5 expression in the group treated with POM-NVs compared to the control group (Fig. 4A).

To further explore the potential of POM-derived nanovesicles in inhibiting BMP5 signaling, we conducted in vitro experiments using the BPH1 cell line (Fig. 4B). BPH1 cells were treated with POM-NVs, followed by real-time PCR analysis of relative BMP5 expression (Fig. 4B). As a positive control, we also treated BPH1 cells with the BMP5 inhibitor LDN 193189^13^. Remarkably, POM-NVs treatment resulted in a significant decrease in BMP5 signaling, surpassing the effect observed with the BMP5 inhibitor.

### Multiple signaling pathways are modulated by POM-derived vesicles in BPH1 cells

**Figure 5:**
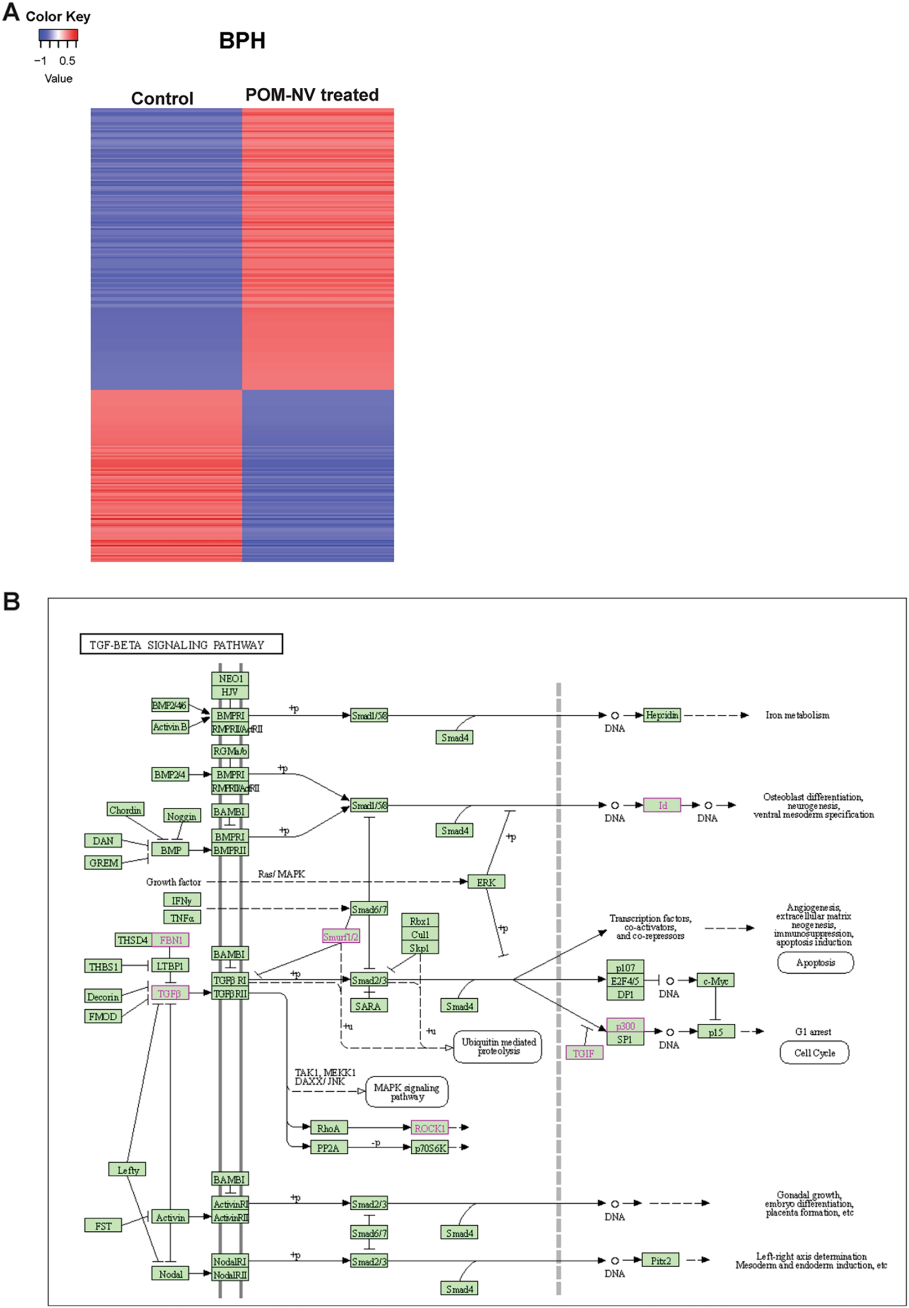
Multiple signaling pathways are modulated by POM-NVs in BPH1 cells. (**A**) Heat map of significantly altered genes in control/POM-NVs treated BPH1 cells. BPH1 cell line was cultured in exosome-depleted media and treated with 10^8^ POM-NVs for 2 days. Following treatment, cells were harvested, total RNA isolated and libraries were generated using TruSeq Stranded mRNA Library prep kit (Illumina). The libraries were pooled and run on the NextSeq500 sequencing system. (**B**) KEGG pathway analyses of significantly altered genes in TGF-β signaling pathway in POM-NVs treated BPH1 cells as compared to control cells.

To gain further insights into the mechanistic basis of the observed effects of POM-derived nanovesicles in BPH, we conducted next-generation RNA sequencing in BPH1 cells treated with POM-NVs (10^8^ particles) for 2 days, comparing them to the control group. The RNA sequencing was performed on the NextSeq500 sequencing system. Our analyses revealed significant dysregulation of a set of 539 genes, with 348 genes upregulated and 191 genes downregulated in the POM-NVs treated BPH1 cells compared to the controls (Fig. 5A and Supplementary Table S1). Among the top upregulated genes were protocadherin 19 (PCDH19), microtubule actin crosslinking factor 1 (MACF1), and 2-hydroxy-3-methylglutaryl CoA synthase 1 (HMGCS1). The top downregulated genes included inhibitor of DNA binding 1 (ID1), serpin family B member 3 (SERPINB3), and keratin 1 (KRT1) (Supplementary Table S1).

Further pathway analyses using KEGG (Kyoto Encyclopedia of Genes and Genomes)^26–28^ showed that POM-NVs significantly affected three pathways: TGF-β signaling (Fig. 5B), genes involved in focal adhesion (Supplementary Fig. S1A), and adherens junction (Supplementary Fig. S1B). In the TGF-β signaling pathway (Fig. 5B), components such as TGF-β2, Fibrillin 1 (FBN1), E1A-binding protein 300, ROCK1, and Smad specific E3 ubiquitin ligase 2 (SMURF2) were increased, while TGF-β-induced factor homeobox 1 (TGIF1), Inhibitor of DNA binding 3 (ID3), and Inhibitor of DNA binding 1 (ID1) were significantly decreased in the POM-NVs treated BPH1 cells compared to the control group. In the focal adhesion pathway, genes such as laminin subunit alpha 3 (LAMA3), fibronectin 1 (FN1), insulin-like growth factor 1 receptor (IGF1R), tenascin C (TNC), epidermal growth factor receptor (EGFR), rho-associated, coiled-coil-containing protein kinase 1 (ROCK1), and integrin subunit beta 6 (ITGB6) were increased, while genes including Rho GTPase Activating Protein 35 (ARHGAP35), β-Actin (ACTB), Myosin Light chain 12A (MYL12A), and Guanine nucleotide exchange factor VAV3 (VAV3) were significantly decreased (Supplementary Fig. S1A). Interestingly, we observed decreased expression of actin cytoskeletal genes upon POM-NVs treatment (Supplementary Fig. S1A). Notably, the prominent adherens junction gene increased by POM-NVs was nectin cell adhesion molecule 3 (NECTIN3) (Supplementary Fig. S1B and Supplementary Table S2).These findings indicate that POM-NVs impact multiple signaling pathways, including TGF-β signaling, focal adhesion, and adherens junctions, which may contribute to their therapeutic effects in BPH.

### Pomegranate juice-derived nanovesicles reverse TGF-β mediated EMT in BPH1 cells

**Figure 6:**
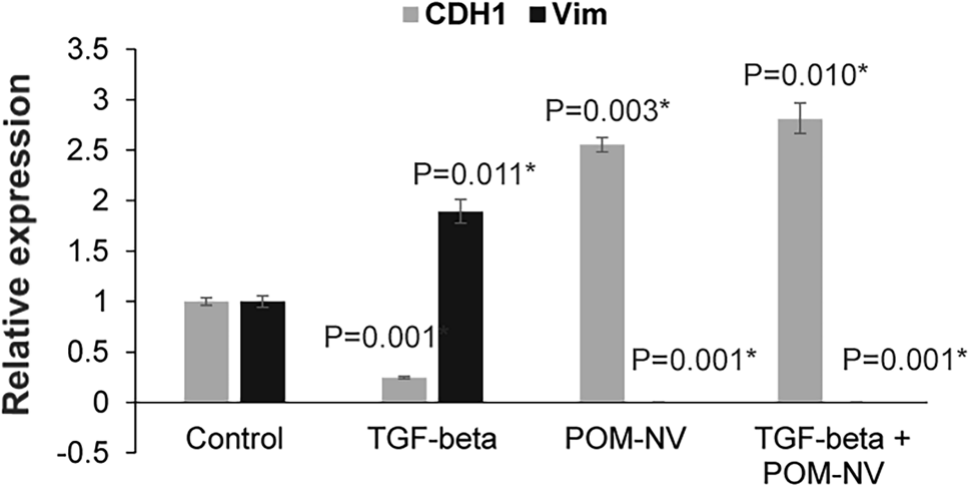
Pomegranate juice-derived nanovesicles reverse TGF-β mediated EMT in BPH1 cells. Real time PCR based analyses of E-cadherin (*CDH1*) and Vimentin (*VIM*) in control BPH1 cells or BPH1 cells treated with TGF-β/POM-NVs/TGF-β + POM-NVs. GAPDH was used as an endogenous control.

Since it has been reported that BPH is associated with TGF-β mediated EMT via activation of Smad signaling^12^, we sought to investigate whether POM-NVs possess the ability to reverse the BPH phenotype induced by TGF-β-mediated signaling. To examine this, we treated the BPH1 cell line with TGF-β in culture and performed real-time PCR-based analyses of effector genes E-cadherin (CDH1) and Vimentin (VIM) (Fig. 6). As expected, TGF-β treatment resulted in decreased CDH1 expression and concurrent increase in VIM expression, consistent with the induction of EMT. However, treatment of BPH1 cells with POM-NVs led to increased CDH1 expression and decreased VIM expression, suggesting that these nanovesicles induce reversal of EMT and promote epithelial states. Moreover, when BPH1 cells were treated with TGF-β in the presence of POM-NVs, the TGF-β-induced EMT was reversed, as evidenced by the induction of CDH1 expression and decreased VIM expression. These findings strongly support the potential of POM-derived nanovesicles in reversing the BPH phenotype by inhibiting TGF-β-mediated EMT.

### Mass spectrometric analyses of pomegranate-derived nanovesicles’ proteome

**Figure 7:**
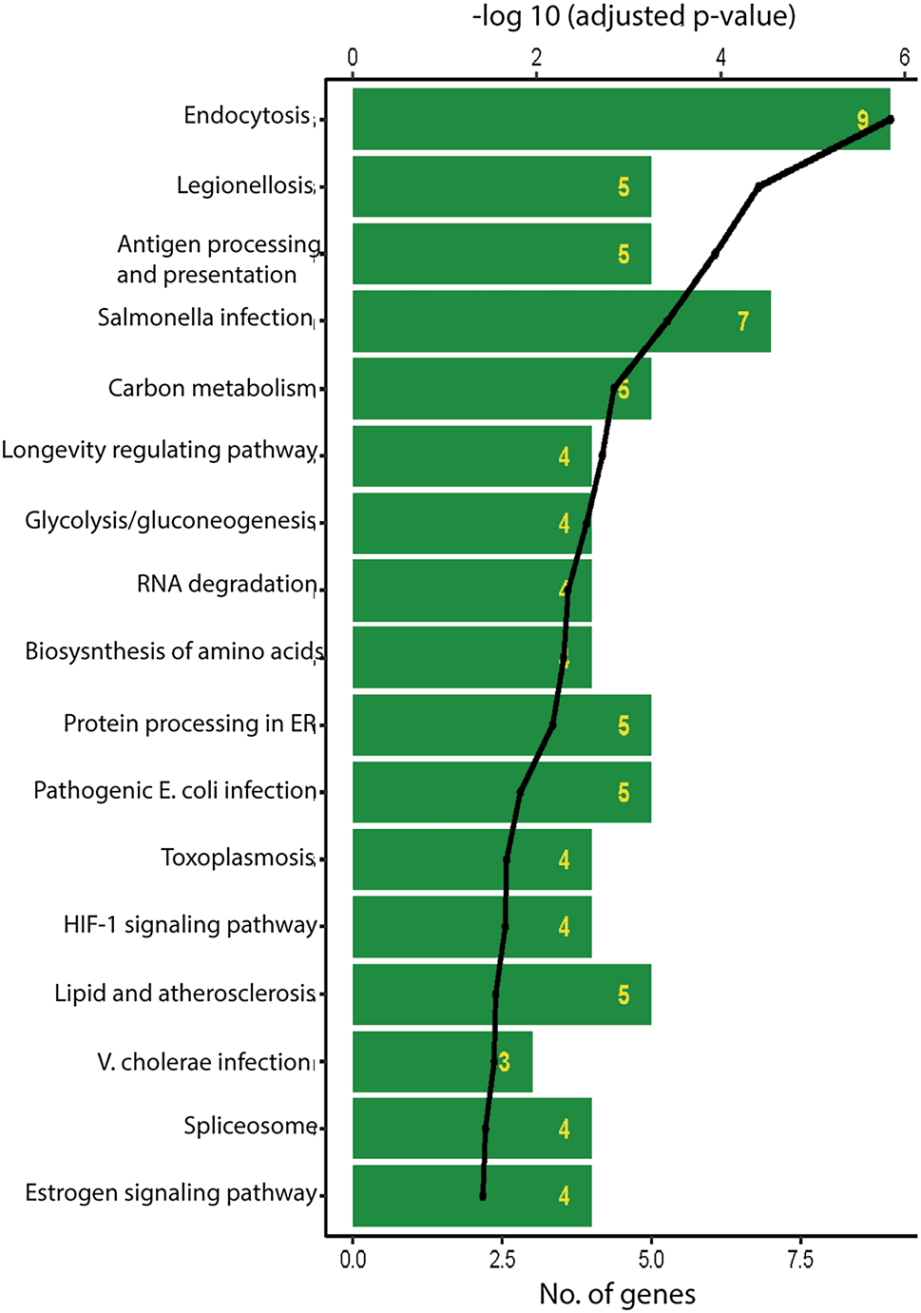
Mass spectrometric analyses of pomegranate-derived nanovesicles’ proteome. We probed the proteome of POM-NVs by mass spectrometric analyses on an Orbitrap Fusion tribrid mass spectrometer. Shown is the functional enrichment analysis for the identified homologous proteins against KEGG database.

Considering the promising therapeutic effects of POM-NVs in BPH, we aimed to further characterize these vesicles by conducting mass spectrometric analyses to identify their protein content (Fig. 7). Through this analysis, we identified a set of 1841 proteins with a peptide count of at least two (Supplementary Table S3). Among the highly abundant proteins, we observed plasma membrane ATPases, heat shock cognate 70 protein (Hsc70), and 14-3-3 like proteins. POM-NVs also contained proteins involved in reactive oxygen species (ROS) signaling, such as phospholipase, ascorbate peroxidase, glutathione-S-transferase, and annexin. Additionally, the vesicles contained membrane trafficking proteins, including syntaxins like syntaxin-52 isoform X2, syntaxin 121-like, syntaxin 22-like, and syntaxin-71-like.

Furthermore, we performed BLASTP analysis by searching the 143 most abundant peptides from the POM-NVs proteome against human proteins. This analysis identified a set of 75 homologous proteins, including ADP ribosylation factors (ARF3, ARF1, ARF5, ARF4, ARF6), heat shock proteins (HSP70 like, HSP70 protein 1A, HSP70 protein 6, HSP70 protein 2, HSP70 protein 1A, HSP70 protein 8), and actin (beta-actin, gamma actin, alpha actin-1, alpha-actin-3), among others. Pathway analyses (Supplementary Table S3) revealed the representation of proteins involved in various cellular processes, including endocytosis, antigen processing and presentation, carbon metabolism, glycolysis/gluconeogenesis, RNA degradation, biosynthesis of amino acids, protein processing in the endoplasmic reticulum, HIF-1 signaling pathway, estrogen signaling pathway, and gluconeogenesis (Fig. 7). Additionally, proteins associated with various infections, such as Legionellosis caused by *Legionella* bacteria, *Salmonella* infection, and pathogenic *E. coli* infection, were also identified (Supplementary Table S3).

## Discussion

We report here to our knowledge for the first time, potential of POM-NVs for effective therapy of BPH, using a clinically relevant human xenograft BPH model. Our data suggests that POM-NVs can be exploited as an effective, low cost and safe BPH therapeutic. Furthermore, this therapeutic agent may impact BPH pathobiology. Importantly, POM-NVs resulted in downregulation of BMP5 signaling in vivo in a BPH xenograft model and in vitro in the BPH1 cell line. Recent studies have shown an elevation in BMP5 signaling as a cardinal alteration driving BPH^6^. BMP5 promotes cell proliferation and the EMT process via phosphorylation of Smad1/5/8 in BPH^13^. BMP5 inhibitor LDN 193189 has shown promising therapeutic potential in BPH^13^. Interestingly, in BPH1 cell line, the degree of apoptosis induction with POM-NVs was more than that with LDN 193189. Furthermore, POM-NVs inhibited the viability of BPH1 cells at levels similar to BMP5 inhibitor. The progression of BPH is facilitated by EMT that leads to decreased E-cadherin expression and accumulation of mesenchymal-like cells derived from the prostatic epithelium and the endothelium^10–12^, mediated by TGF-β that acts via activation of Smad signaling^12^. Importantly, POM-NVs could reverse the BPH phenotype conferred by TGF-β treatment in BPH1 cell line and in vivo in BPH xenograft model. These data validated the therapeutic ability of POM-NVs in reversing BPH.

One of the obstacles to the development of effective BPH treatments is the lack of a practical and reliable study model. In this study, we generated a nude mouse BPH xenograft model using human clinical BPH tissues and employed it to assess the therapeutic potential of POM-NVs. H & E staining and AR staining showed that this model is a reliable BPH model that preserves the critical cellular architecture of xenografted human BPH tissues. Our data support that primary tissue xenografts of fresh clinical BPH tissues provide a valuable model for studying the regulatory effects of systemically administered drugs. Our in vivo studies with this model validated the potential of POM-NVs to reverse EMT and ameliorate BPH phenotype.

BPH pathophysiology is poorly known, preventing targeted therapy. Importantly, our sequencing analyses of POM-NVs treated BPH1 cell line revealed novel molecules/signaling pathways that can be potentially targeted in BPH. In addition to TGF-β pathway, focal adhesion and adherens junctions were modulated by POM-NVs suggesting that modulation of these cellular processes may be of therapeutic value in BPH. Interestingly, Inhibitor of DNA binding protein 1 (ID1) and Inhibitor of DNA binding protein 3 (ID3) were prominently downregulated by treatment with POM-NVs. These helix-loop-helix (HLH) proteins lack a DNA binding domain and can heterodimerize with other HLH proteins with DNA binding domains, thereby titrating these proteins away from their DNA binding sites. These proteins have been implicated in regulating a variety of cellular processes including growth, senescence, differentiation, apoptosis and angiogenesis^29^. However, the role of these proteins in BPH have not been studied. Future studies focusing on understanding the mechanistic role of these proteins in BPH pathogenesis and potentially targeting these proteins directly for BPH therapy are warranted.

The chemopreventive and chemotherapeutic effects of pomegranate juice have been well studied in prostate cancer^21^. However, its effects on BPH have not been well explored. Ammar et al. reported the protective effects of pomegranate fruit extract in preventing testosterone-induced BPH in a rat model^25^. To our knowledge, we report for the first time, the isolation and characterization of nanovesicles from pomegranate juice and identified the therapeutic potential of these nanovesicles in BPH by systemic administration in vivo. Plant-derived vesicles are currently understudied and uncharacterized^19^. An interesting study reported that plant-derived EVs are involved in plant-pathogen interactions wherein plant-derived EVs are uptaken by fungal cells to inhibit its growth^30^. In another study, EVs were isolated from apoplastic fluids of *Arabidopsis thaliana* leaves and were reported to be an important component of plant immune responses^31^. In congruence with these studies, our proteomic analyses of POM-derived nanovesicles showed enrichment of proteins involved in various infections such as pathogenic *E. coli* infection suggesting that one of the functions of POM-NVs may be to protect the plant from infections. Presence of proteins involved in various infections can have potential anti-inflammatory, immune modulating and tissue remodeling effects in BPH. These proteins may possess anti-inflammatory properties that can potentially modulate the inflammation associated with BPH. Furthermore, immune-related proteins found in the POM-NVs may influence the local immune response within the prostate, potentially promoting a more balanced immune response against BPH. Some of these immunity-related proteins have been implicated in tissue remodeling processes, which may contribute to the restoration of normal tissue architecture and alleviation of the obstruction caused by BPH. However, it is important to note that these potential effects are speculative, and further research is necessary to determine the precise effects of these proteins in BPH. Further, proteomic analyses of POM-NVs showed enrichment of vesicle-related proteins such as membrane trafficking syntaxins, plasma membrane ATPases and heat shock cognate 70 proteins. The presence of these proteins in plant-derived vesicles has been reported before^31^.

Pomegranate is known to be rich in anti-oxidants. Pomegranate juice contains anthocyanins, ellagic acid derivatives, and hydrolyzable tannins such as punicalagins^21,32^. The vesicles derived from pomegranate juice is rich in anti-oxidants that could contribute to the observed therapeutic effects against BPH. Proteomics analyses of POM-NVs showed an enrichment of proteins involved in reactive oxygen species (ROS) signaling suggesting that these vesicles may help modulate levels of ROS and alleviate cellular oxidative stress. Interestingly, POM-NVs contained several 14-3-3 like proteins. These proteins are associated with metabolic regulation in plants and mammals^33^ and act as master switches in insulin signaling, mammalian target of rapamycin (mTOR), and AMP-dependent kinase signaling pathways^33^. These pathways are recognized as crucial contributors to the development of BPH. 14-3-3 proteins are also involved in modulation of cell death, cell cycle and cytoskeletal dynamics^33^. However, the role of 14-3-3 proteins in BPH pathogenesis is not defined. Future mechanistic studies on these adaptor proteins in BPH are needed.

In conclusion, we report here for the first time (i) a novel, cheap, scalable, pomegranate juice-derived nanovesicle based therapeutic strategy for targeting BPH using a clinically relevant human BPH xenograft model. (ii) the proteome of POM-derived nanovesicles. Considering the large incidences of BPH among men and the associated economic burdens, our study has important implications in the field and can potentially improve the clinical management of BPH.

## Methods

### Cell lines and cell culture

Immortalized non-transformed prostate epithelial cell line (BPH1)^34^ was maintained in RPMI 1640 media supplemented with 5% FBS, and 1% penicillin/streptomycin. Cells were maintained in an incubator with a humidified atmosphere of 95% air and 5% CO_2_ at 37 °C.

### Isolation of nanovesicles from pomegranate juice

Fresh pomegranate fruit (cultivated) was procured from a local grocery store from which pomegranate juice was extracted. Pomegranate juice was extracted from whole fruit by rolling the pomegranate with hands on sterile tabletop to soften the fruit followed by squeezing of the juice into a beaker. POM juice was subjected to an initial centrifugation at 500*g* for 10 min. Next, the obtained supernatant was centrifuged at 2000*g* for 20 min followed by centrifugation at 10,000*g* for 30 min. Next, the supernatant was passed through a 0.2 µm filter. The supernatant was then subjected to ultracentrifugation at 100,000 g for 2 h in a Beckman Coulter ultracentrifuge (Indianapolis, IN) with 45T rotor. The obtained pellet of nanovesicles was resuspended in sterile PBS. Recently, International Society of Extracellular Vesicles (ISEV) recommended the term ‘plant-derived nanovesicles’ for vesicular fractions obtained from plant tissues^19^. In keeping with these recommendations, we refer to these vesicles as ‘POM-derived nanovesicles’ or ‘POM-NVs’ in this study. We have submitted all relevant data of our experiments to the EV-TRACK knowledgebase (EV-TRACK ID: EV230599)^35^.

### In vivo xenograft studies

Animal studies were approved by Augusta University Institutional Animal Care and Use Committee and were performed in accordance with institutional guidelines under an approved protocol. Human clinical BPH tissues (transition zone) were collected from BPH patients by transurethral resection of the prostate under an approved Institutional Review Board (IRB) protocol with informed consent. In the operative suite, BPH tissues were placed in RPMI media immediately after resection. Tissues were then transported to the laboratory and sliced into 1mm sections using a standard scalpel. *nu/nu* male mice (6 weeks old, Charles River Laboratories, Colbert, GA) underwent bilateral orchiectomy and placement of a 12.5 mg testosterone pellet (Innovative Research of America, Sarasota, Florida) ventrally. Tissue slices (4 per mouse) were covered in Matrigel and implanted subcutaneously under the dorsal skin. Xenografts were allowed to establish for 4 weeks. Once established, animals were categorized into control/test group (n = 4/group). Test mice were administered 10^9^ POM-NVs via tail vein (in PBS) twice a week for 3 weeks. Controls included xenografts treated with PBS for the same time period at the same frequency. After 3 weeks, mice were sacrificed and xenografts were harvested and analyzed. Xenografts were embedded, sectioned and stained with H&E. Animal studies are reported in accordance with the ARRIVE guidelines.

### Statistics

All quantified data represents an average of triplicate samples or as indicated. Experiments with cell lines included at least three biological replicates. Data are represented as mean ± S.E.M or as indicated. Statistical significance between groups was assessed by Student's t-test. Results were considered statistically significant at P ≤ 0.05.

## Supplementary Information

Supplementary Figure S1.

Supplementary Information.

Supplementary Table S1.

Supplementary Table S2.

Supplementary Table S3.

## Data Availability

All data generated or analyzed during this study are included in this article and its supplementary information files.
