# Supplementary figures and images for: Therapeutic potential of pomegranate juice-derived nanovesicles in nude mouse benign prostatic hyperplasia (BPH) xenograft model
Source: Sci Rep. 2023 Aug 1;13:12427. doi: 10.1038/s41598-023-39511-w (PMC10394011; doi:10.1038/s41598-023-39511-w)

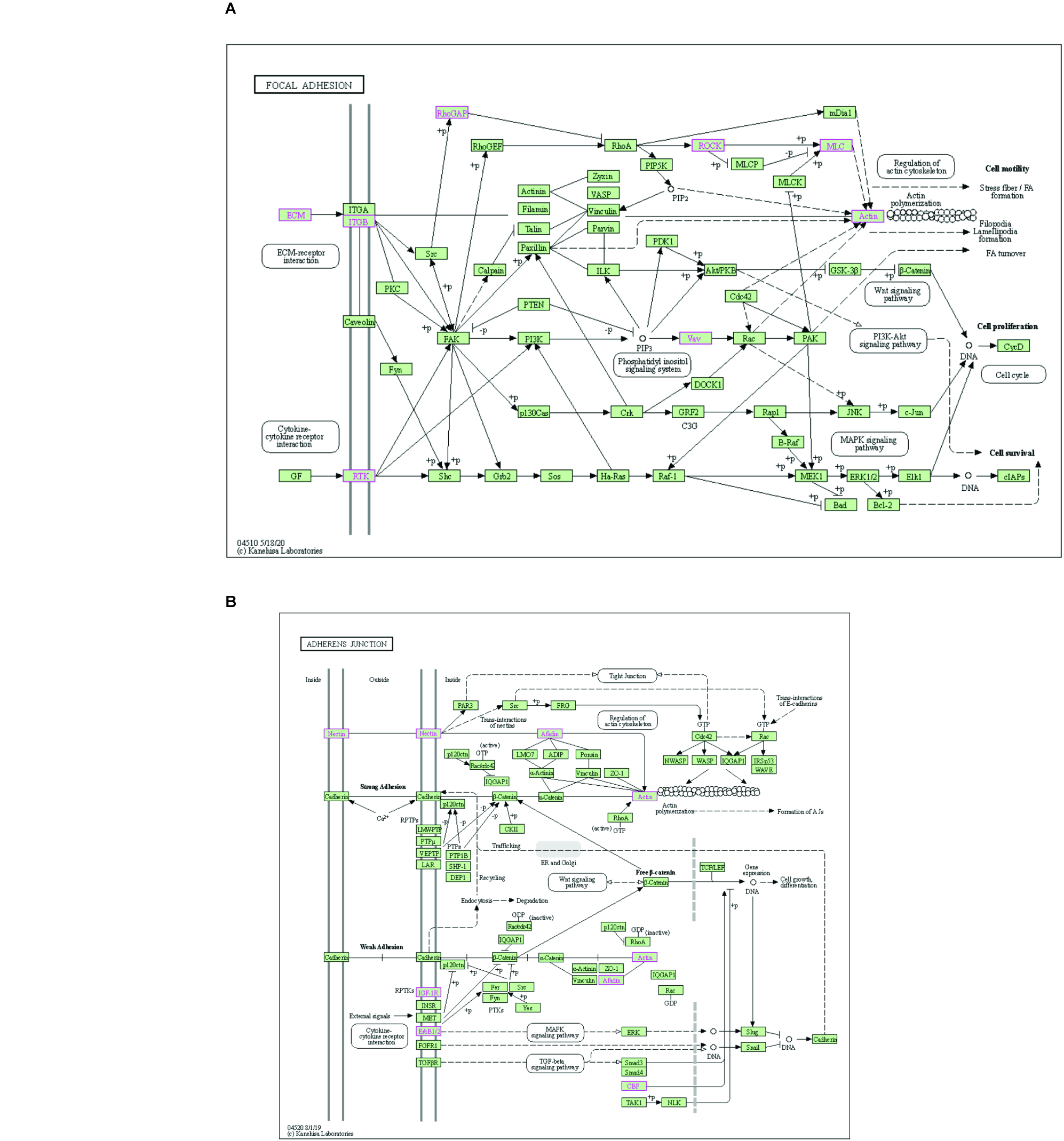

Supplement: Supplementary file 1 — Supplementary Figure S1. [file 41598_2023_39511_MOESM1_ESM.tif]
