# Supplementary material for: Therapeutic potential of pomegranate juice-derived nanovesicles in nude mouse benign prostatic hyperplasia (BPH) xenograft model
Source: Sci Rep. 2023 Aug 1;13:12427. doi: 10.1038/s41598-023-39511-w (PMC10394011; doi:10.1038/s41598-023-39511-w)
Supplement: Supplementary file 2 — Supplementary Information. [file 41598_2023_39511_MOESM2_ESM.docx]

**Therapeutic potential of pomegranate juice-derived nanovesicles in nude mouse benign prostatic hyperplasia (BPH) xenograft model**

Sreekumar at al.

**SUPPLEMENTAL METHODS**

# Treatment of BPH1 cells

BPH1 cell line was cultured in exosome-depleted media and treated with 10^8 POM-NVs or PBS for 2 days. For TGF-β treatment, BPH1 cells were plated with 5nM recombinant TGF-β (Sigma Aldrich, St. Louis, MO) and treated for 2 days. For combined treatment, cells treated with 5nM recombinant TGF-β were additionally treated with 10^8 POM-NVs for 2 days. For BMP5 inhibitor, BPH1 cells were treated with 2 micromolar LDN 193189 (Catalog no. HY 12071A) (MedChemExpress, Monmouth Junction, NJ) for 2 days.

# Nanovesicle quantitation and size determination by Nanoparticle Tracking Analyses

To confirm the integrity of POM-NV preparations, the purity of nanovesicles was verified by evaluation of particle size and concentration using Nanoparticle Tracking Analysis (NTA). A NanoSight NS300 instrument (Malvern Instruments, Malvern, UK) equipped with a 405nm laser-equipped sample chamber was employed as per manufacturer’s instructions. Prior to sampling, the sample solutions were homogenized by vortexing and serially diluted with 0.2 μm-filtered 1x PBS to a final dilution of 1:1000. The sample chamber was filled with PBS diluted sample and the shutter and gain were manually adjusted for optimal detection. Each sample was analyzed with NTA, 3.4 software and each analysis consisted of three-five 30-s .avi (audio video interleaved) file recordings.

# Transmission Electron Microscopy

To further validate the nanovesicle preps, transmission electron microscopy (TEM) was performed on the purified EVs as described in ^31^. Briefly, 5 µl of exosome suspension was placed on a Formvar/carbon 200 mesh copper grid (Electron Microscopy Sciences, Hatfield, PA) and allowed to dry. 5ul of 2% aqueous uranyl acetate was then added to the grid and incubated for 5 minutes before excess uranyl acetate was removed and grids were viewed in a JEOL 1400Flash transmission electron microscope (JEOL Inc., Peabody MA) at 120kV. Images were acquired with a Gatan OneView digital camera (Gatan Inc., Pleasanton CA) using Digital Micrograph software.

# Cellular viability assay

For cell viability assay, BPH1 cells were seeded in exosome-depleted RPMI media in 96-well microplates at a density of 5x10^3^ cells per well 24 hours before treatment. After treatment with

10^8 POM-NVs for 48 hours, cell viability was determined by using the CellTiter 96 AQueous One Solution Cell Proliferation Assay kit (Promega, Madison, WI) according to the manufacturer’s protocol. Absorbance at 490 nm was measured with a multi mode reader (Synergy HTX, Santa Clara, CA).

# Apoptosis assay

Control BPH1 cells or BPH1 cells treated with an increasing concentration of POM-NVs (10^7 particles and 10^8 particles, respectively) for 48 hours were harvested. After PBS wash, cells were stained with Annexin V-FITC/7-AAD (Beckman Coulter, Indianapolis, IN) according to manufacturer’s instructions. Stained cells were analyzed on BD Cytoflex (Beckman Coulter, Indianapolis, IN) within 1 hour.

# Mass spectrometric analyses of nanovesicles

Enriched nanovesicle samples were dried by lyophilization. 100 µl of freshly made 50mM ammonium bicarbonate buffer with 0.1% (w/v) heat-labile RapiGest SF Surfactant (Waters) and 10mM dithiothreitol was added into sample tube to resuspend the vesicle sample and reduce the disulfide bonds at 60ºC for 30 minutes. The samples were then alkylated by iodoacetamide in the dark for 30 minutes, followed by digestion for 16 hours using trypsin (Cat no. 90057) (Thermo Scientific, Waltham, MA) at 37°C. Trifluoroacetic acid was added to the sample tube to a final concentration of 0.1% (v/v) to stop the digestion. The samples were then incubated at 37°C for 40 minutes to cleavage the detergent. The samples were cleaned using C18 spin column (Cat no. 744101) (Harvard Apparatus, Holliston, MA) and then lyophilized. Digested peptide samples were first reconstituted into 60 µl of 2% acetonitrile with 0.1% formic acid and then analyzed on an Orbitrap Fusion tribrid mass spectrometer (Thermo Scientific, Waltham, MA), coupled with an Ultimate 3000 nano-UPLC system (Thermo Scientific, Waltham, MA). Six microliters of peptide sample was first trapped on a Pepmap100 C18 peptide trap (Thermo Scientific, 5um, 0.3X5mm) and then washed at 20 µl/min using 2% acetonitrile with 0.1% formic acid for 10 minutes. Next the cleaned peptides were washed off the trap and further separated on a Pepman 100 RSLC C18 column (Thermo Scientific, 2.0 um, 75-μm × 150-mm) at 40°C using a gradient of between 2% to 40% acetonitrile with 0.1% formic acid over 120 min at a flow rate of 300 μl/min. LC-MS/MS analysis were performed using data-dependent acquisition (DDA) in positive mode with the Orbitrap MS analyzer for precursor scans at 120,000 FWHM from 400 to 1600 m/z using quad isolation and the ion-trap MS analyzer for MS/MS scans at top-speed mode (3-second cycle time). Higher-energy collisional dissociation (HCD) was used to fragment the precursor peptides with a normalized energy level of 30%. The mass spectrometry proteomics data have been deposited to the ProteomeXchange Consortium via the PRIDE [1] partner repository with the dataset identifier PXD043608.

# Protein database search

Raw MS and MS/MS spectrum for each sample were filtered and processed using the Proteome Discoverer software by Thermo Scientific (v1.4) and then submitted to SequestHT search algorithm against the Uniprot *Punica Granatum* database (10 ppm precursor ion mass tolerance: 10ppm, product ion mass tolerance: 0.6 Da, static Carbamidomethylation of +57.021 Da for cysteine and dynamic oxidation of +15.995 Da for methionine). Perculator PSM validator algorithm was used for peptide spectrum matching validation and false discovery rate estimation.

Most abundant peptides from POM-NVs mass spectrometry data were obtained with threshold of

10 peptide spectra match (PSM). These peptides were searched against human proteins using BLASTP. E-value threshold of 0.01 was used for homologous protein search. Functional enrichment analysis was performed for the homologous proteins against database including gene ontology, KEGG, and TRANSFAC using gprofiler2 package in R.

**RNA isolation and quantitative real time PCR**

Cellular RNAs were prepared using a RNeasy kit (Qiagen, Germantown, MD) as per manufacturer’s instructions. The quantity and quality of the RNA was determined by an Agilent

Bioanalyzer 2100 (Agilent Technologies, Santa Clara, CA) as per the manufacturer’s instructions.

Mature mRNAs were assayed using the TaqMan Gene Expression Assays (Life Technologies,

Rockville, MD) in accordance with the manufacturer's instructions. TaqMan assays used were BMP5 (assay ID Hs00234930_m1), and GAPDH (Hs99999905_m1). The comparative Ct method was used to calculate the relative changes in gene expression on the Step OnePlus Real Time PCR System.

**mRNA sequencing**

RNA purity and concentration were evaluated by spectrophotometry using NanoDrop ND-1000

(ThermoFisher, Waltham, MA). RNA quality was assessed by the Tape Station (Agilent Technologies) and assured of a RNA Integrity Number (RIN) greater than 7. Total RNA samples were processed for cDNA library preparations using TruSeq Stranded mRNA Library prep kit (Illumina, San Diego, CA). Briefly, mRNA was captured using oligo-dT beads from 500 ng of total RNA. Following purification, the mRNA was fragmented into small segments, 300 - 350 bp in size, and converted to cDNA fragments. These cDNA fragments then had the addition of a single ‘A’ base and subsequent ligation of the adapter. The products were purified and enriched with PCR to create the final cDNA library. The prepared library was further examined by Tape Station and Qubit (Thermofisher, Waltham, MA) to test for quality and quantity, respectively. The libraries were pooled and run on the NextSeq500 sequencing system using a 75-cycle paired-end protocol. Quality control of the raw reads were performed by FastQC, and adapters were removed using cutadapt version 4.1. Trimmed reads were aligned to the human reference genome of hg38 and quantified using STAR version 2.7.0. For altered genes with fold change threshold of 10, enrichment analysis was carried out against database including gene ontology, KEGG, and TRANSFAC using gprofiler2 package in R.

# Immunohistochemistry

Immunostaining was done on formalin-fixed, paraffin-embedded BPH xenograft tissues. Slides were deparrafinized, antigen retrieval was carried out by microwaving the slides in 10 mmol/L sodium citrate buffer followed by overnight incubation with anti-E-cadherin antibody (Cat no. 3195) (Cell Signaling, Danvers, MA), AR antibody (Cat no. 5153) (Cell Signaling, Danvers, MA) or TGF-β antibody (Cat no. 3711) (Cell Signaling, Danvers, MA). Following washes with PBS, anti-rabbit secondary antibody was added and slides were incubated for 1 hour at room temperature. Slides were washed with PBS and developed with DAB staining kit (Immpact, Vector Laboratories, Newark, CA) following manufacturer’s instructions. The slides were counterstained with hematoxylin.

**Fractionation on iodixanol gradient**

A 6-18% iodixanol gradient was made from iodixanol density gradient medium (Sigma) in dry swinging bucket ultracentrifuge tube starting with 6% and ending in 18% 30. POM-EVs in PBS were added on top of the layered gradient and samples were ultracentrifuged at 100,000 g for 90 min. The fractions were collected and analyzed on Nanosight NS300 instrument. Also, the fractions were separately ultracentrifuged to spin down the fractionated EVs.

**SUPPLEMENTAL FIGURE LEGEND**

**Fig. S1 Focal adhesion and adherens junction are modulated by POM-derived vesicles in**

**BPH1 cells**

1. KEGG pathway analyses of significantly altered genes in focal adhesion pathway in POM-

NVs treated BPH1 cells as compared to control cells.

1. KEGG pathway analyses of significantly altered genes in adherens junction pathway in POM-NVs treated BPH1 cells as compared to control cells.

**SUPPLEMENTAL TABLE LEGENDS**

# Table S1 List of genes regulated by POM-NVs in BPH1 cells

A set of 539 genes are dysregulated by POM-NVs treatment in BPH1 cells as compared to control as determined by next generation RNA sequencing on the NextSeq500 sequencing platform.

# Table S2 KEGG (Kyoto Encyclopedia of Genes and Genomes) pathway analyses of altered genes by POM-NVs in BPH1 cells

KEGG pathway analyses of altered genes by POM-NVs in BPH1 cell line as determined by next generation RNA sequencing in BPH1 cells as compared to control (no treatment).

# Table S3 Mass spectrometric analyses of pomegranate-derived nanovesicles’ proteome

Sheet 1: Mass spectrometric analyses identified a set of 1841 proteins in POM-NVs with a peptide count of at least two.

Sheet 2: 143 most abundant peptides from POM-NVs proteome were searched against human proteins using BLASTP. Listed are identified homologous proteins.

Sheet 3: Functional enrichment analysis was performed for the homologous proteins against database including gene ontology, KEGG, and TRANSFAC.

**REFERENCES**

1. Perez-Riverol Y et al. The PRIDE database resources in 2022: A Hub for mass spectrometry-based proteomics evidences. Nucleic Acids Res 50(D1):D543-D552 (PubMed ID: 34723319). 2022.
